# Proceedings of 4th National Conference of Pakistan Association of Medical Editors held at Khyber Medical University, Peshawar (March 3-4, 2018)

**DOI:** 10.12669/pjms.342.15180

**Published:** 2018

**Authors:** Shaukat Ali Jawaid

PESHAWAR: Pakistan Association of Medical Editors (PAME) organized its fourth National Conference at Khyber Medical University Peshawar from March 3-4^th^ 2018. Prof. Arshad Javed Vice Chancellor of Khyber Medical University was the host and all this became possible due to his and his team’s keen interest. Prof. Arshad Javed, it may be mentioned here is himself member of PAME and as Chief Editor of Journal of Postgraduate Medical Institute for quite some time, has been the moving spirit for promoting the discipline of medical journalism in Khyber PK. Prof. Akhtar Sherin Chief Editor of Khyber Medical University Journal and Dr. M. Irfan were members of the organizing committee.

## Starting a New Journal and maintaining quality of publication

The conference started on the morning of March 3, 2018. The first session was devoted to Starting a New Journal and Maintaining quality of publication. This was moderated by **Dr. Jamshed Akhtar** Editor of Journal of College of Physicians & Surgeons Pakistan. He also made the first presentation and talked about scope of the journal and need assessment. Many journals, he opined had inexperienced editorial board and quality of the manuscripts they publish was also questionable. Some journals face paucity of manuscripts. Bypassing the editorial process and draconian rules by the authorities were some of the challenges faced by the medical journals in Pakistan.

Editors must get experience, show commitment and dedication-Jamshed Akhtar

Higher Education Commission, Government of Pakistan, he said has recognized various journals in different categories which are as under:

W Category-4

X Category-5

Y Category-16

Z Category-11

Derecognized-20

The question arises should we upgrade the existing journals or start a new journal? The criteria used for recognition of journals by the regulatory bodies, he opined, are also questionable. PM&DC has recognized seventy two journals but what are their criteria is not known. Universities, professional specialty organizations, medical colleges, individual publishers are publishing journals and their aims and objectives are different. There is a need for renowned medical publishers. The Mission, Goal and objectives of the journal should be clearly defined. Some of the subspecialty journals, he said, were quite successful and have good Impact Factor as well. Name of the journal also matters, he remarked. What a journal does not accept should be clearly stated on the journal website. Before starting a journal, one must get experience as it is not a part time job. Show commitment and dedication. Courses for training of editors are available on WAME website and some other sources as well. PAME has also been considering starting certificate course in medical journalism, some discussions were also held but it could not be materialized so far due to various reasons.

Editor should be ethical, professional, they have to bear accusations, show patience and must be supportive -Fatema Jawad

**Dr. Fatema Jawad** Chief Editor of JPMA spoke about editorial freedom. She opined that Editor should be ethical and professional; journal must have a transparent policy, have professional relationship and never misuse their position. Editors have to bear accusations, show patience and must be supportive. Editors should have complete independence. Editorial freedom is extremely important. HEC recently de-recognized twenty medical journals and all of them are recognized by the PM&DC. In practical life, it is seen that the Editors of journals published by universities cannot take decisions; they even cannot have meetings if the Vice Chancellor is not available. Sociology journals enjoy more freedom. Honesty, she opined, was a protective factor against coercion. Editors must be honest, update their knowledge, should be hardworking and ensure transparency, regular publication and high quality should be evident, she added.

**Prof. Saba Sohail** also from the JCPSP talked about In-House assessment- a key step in processing the articles. She pointed out that it must be ensured that all the basic requirements are there, all the mandatory documents have been submitted by the authors, and plagiarism check is a must. One must look at the protocol registration, conflict of interest statement. Do not go for short cut. What type of articles and journal policy as regards categorization is also important? Make sure that English language is good, see if the title is appropriate, the study has a message and it is conveyed effectively. Are the number of authors appropriate, manuscripts has all the important components including conclusions and references before it is accepted for further processing.

Peer review policy adopted by the Journal must be mentioned on the journal website-Akhtar Sherin

**Prof. Akhtar Sherin** Chief Editor of Khyber Medial University Journal talked about Peer Review: Issues and Challenges for a new Journal. Peer review, he said, assures validity, quality. Most of the articles should be peer reviewed and at least one of the reviewers should be external. He also talked about collaborative peer review, third party review before submission by authors to the journal. He then discussed post publication review and who can be a reviewer and how to identify the reviewers. Authors can also suggest reviewers but the Editorial team has to check their credentials. Poor Review is something which one must avoid if one has to maintain quality. Some reviewers do electronic review with track changes. The peer review policy adopted by the Journal must be mentioned on the journal website, he added.

**Prof. Maj.Gen. Muhammad Aslam** Pro VC National University of Medical Sciences and former President of PAME in his presentation highlighted how to maintain quality of the manuscripts. What is given to you, one must read it, correct it. Endurance is needed to maintain quality of manuscripts for a long time. He made it clear that publishing a good quality peer reviewed journal is not a one man job. It is a team work. It requires active contributions by the Editor, Authors, Reviewers, Librarians, Readers, Funding agencies, community and the professional specialty organizations, Accreditation bodies, research organizations, educational institutions. In short it requires multipronged responsibilities, he added.

**Mr. Shaukat Ali Jawaid** Chief Editor Pakistan Journal of Medical Sciences and Secretary of Eastern Mediterranean Association of Medical Editors (EMAME) discussed issues encountered from acceptance to publication. He referred to the pressure from authors to get acceptance letter without realizing that it can only be issued once the peer review and all other formalities have been completed. These manuscripts also need post acceptance editing, and then there is delay in arranging publication charges by the authors, sale and purchase of authorship and gift authorship issues also needs to be tackled effectively. When PDF file is sent for proof reading, some authors do not know how to make corrections, at times the message is not clear and they have to be reminded again and again. Instead of making corrections some authors indulge in rewriting extensively which creates lot of problems requiring reformatting of the manuscript. After corrections the manuscript is published ahead of print on the journal website and it provides yet another opportunity to the authors to see if all the corrections they had marked have been carried out and if not, they can point it out and get it corrected.

Then DOI is generated and as per new rules one can only do that once the manuscript has been published on the journal website as it requires its link. Then comes the page numbering once contents of the whole issue have been finalized. For technical reasons, all the forms have to be equally divided by eight the number of pages on one plate for printing and if this number is not appropriate, one has to either add or delete an article. Sometimes authors ask for withholding publication without realizing that all the publication schedule and page numbers have been finalized. After page numbering, the PDF files are generated again and then published on the journal website. This is followed by computerized page make up, preparing the output for platemaking and then printing, binding. Correspondence author is sent one print copy and if the authors need additional copies, they have to order and pay for it at the time of paying publication charges. Sometimes the authors complain after couple of years or six to eight months that they did not get a printed copy though it was sent but they did not save it. If additional copies are not available they are provided signed and stamped pdf of their manuscript for submission to the PM&DC or other institutions which need it when deciding their cases of promotion.

Authors insist on getting acceptance letter immediately without realizing that it can only be issued once the peer review and all other formalities have been completed- Shaukat Ali Jawaid

**Prof. Nasirudidn Azam Khan**, an eminent physician and former Principal of Khyber Medical College was the Chief Guest in the inaugural session while the founder Vice Chancellor of KMU Prof.Daud Khan graced the occasion as Guest of Honour. The theme of the conference was “Visibility of Pakistani Journals in International Arena: A challenge”. The conference was dedicated to late Dr. Maqbool H. Jafary an eminent physician; founder Chief Editor of Pakistan Journal of Medical Sciences and former President of Eastern Mediterranean Association of Medical Editors (EMAME) in recognition of his services to promote the discipline of Medical Journalism in the Region. This session was moderated by Prof. Akhtar Sherin, former President of PAME and Chief Editor of Khyber Medical University Journal.

**Figure:**
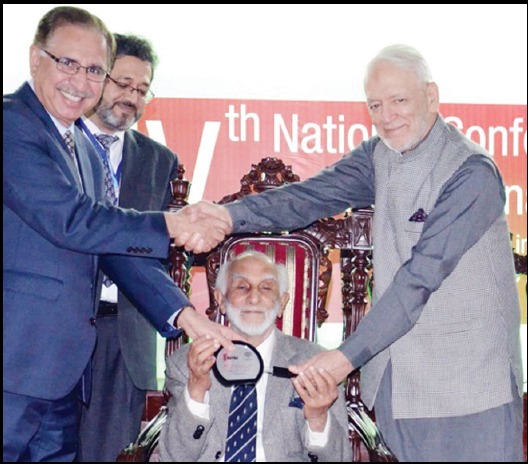
Prof. Arshad Javed alongwith Prof. Daud Khan presenting a Memento to Prof. Nasiruddin Azam Khan former Principal of Khyber Medical College who was the chief guest at the PAME's Fourth National Conference held at KMU Peshawar on April 3, 2018.

In his speech on this occasion Prof. Nasirudidn Azam Khan said that Pakistan has made tremendous progress in the field of medical education and health services if we compare with what we had at the time of independence in 1947. We now have a galaxy of talent and our future is promising. Medical professionals must keep themselves update with latest developments in their respective fields and disseminate knowledge as it is an essential component of research and development.

The conference, Prof. Nasiruddin Azam Khan further stated will highlight the research activity in Pakistan and our accomplishments so far. Research, he opined, was an important weapon which can be used for the benefit of human beings. Professional media has great responsibilities; it must ensure that there are no malpractices which have crept into our professional institutions. Media can also be misused hence it is its responsibility not to publish information which is not useful for the community. It must uphold ethics in publication. There is no doubt that we are faced with lot of difficulties and problems but we must face these challenges and find a solution to these problems, he remarked.

**Prof. Daud Khan** a noted ophthalmologist and founder Vice Chancellor of Khyber Medical University commended the contributions of Chief Guest Prof. Nasiruddin Azam Khan who was one of the most devoted and dedicated teacher of Khyber Medical College in 1968-69. No one ever wanted to miss his lecture. He was an excellent teacher and a mentor. He was very tough but very kind, caring and passionate in examination. I worked as House Officer in his ward and he used to take three rounds daily, one early in the morning, then after attending the Out Patients and then before going home.

As Muslims, Prof.Daud Khan said, we must have Faith in God, and then we have to act on his Commands, preserve that faith and be kind to the community. Referring to the theme of the conference, he said, it was a great challenge. It is a gigantic task. Universities are supposed to produce research which is useful for the local community. He recalled that when University of Baghdad was established, it was known as a House of Wisdom. Medical universities should promote medical education and keep on discovering more and more. Nature, Prof.Daud Khan said has created everything but we need to discover it, publicize it. For application of knowledge, its publication is important. Now we have one hundred seventeen medical colleges in the country. There used to be just one medical college in Khyber PK but now we have eighteen. We need to concentrate on Research and Development. Higher Education Commission has come up with guidelines. For research, access to resources is essential and we need to conduct research which is relevant to our needs. There should be no compromise on quality. He recalled that Gen.W.A.Burki when he was the Federal Health Minister established four institutions on one day i.e. Pakistan Medical & Dental Council, Pakistan Medical Research Council, College of Physicians & Surgeons Pakistan and Jinnah Postgraduate Medical Centre. However, our contributions to research is very disappointing. What we need is a National Commission on Biomedical Sciences which should identify the area of research keeping in view our problems, current population and the future population. We are going to have tremendous increase in elderly population which will have lot of problems; hence we need to look after their healthcare needs. With dedication, devotion and hard work the dream of good quality manuscripts being published in Pakistani medical journals can be realized to come at par with the international standards, he added.

**Figure:**
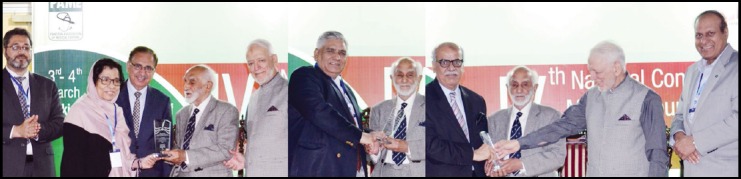
Organizers of PAME's Fourth National Conference held at KMU Peshawar honoured three senior most Medical Editors in recognition of their services to promote the discipline of Medical Journalism in Pakistan. Picture taken on the occasion shows from (L to R) Prof. Fatima Jawad Chief Editor JPMA, Mr. Shaukat Ali Jawaid Chief Editor Pakistan Journal of Medical Sciences and Prof. Maj. Gen.M. Aslam being presented Mementoes by Prof. Nasiruddin Azam Khan along with Prod. Daud Khan and Prof.Arshad Javed VC KMU.

**Prof. Nasir Khokhar** President of PAME in his address said that doctors have too many roles but we need not forget research and medical writing. It is a part of our professional obligations which is also important for our professional development. We can raise our country’s name as well by producing good quality research and Publications. He suggested that we need to involve young doctors in these fields so that they can take over the responsibilities in the days to come.

**Prof. Arshad Javed** Vice Chancellor of Khyber Medical University said that Khyber Medical University Journal has completed Ten Years of its successful publication. When I was told that PAME plans to hold its National Conference, I suggested that it will be a good idea to celebrate our Tenth Anniversary of the KMU Journal by hosting the PAME conference. Members of PAME, Prof.Arshad Javed remarked are a group of selfless professionals. They are doing a commendable job and it will strengthen the research culture in Pakistan.

Speaking about the role of regulatory bodies like the PM&DC and the HEC, Prof. Arshad Javed said that most of the editors of medical journals are not happy with them. Now the PM&DC is going through transformation and at the recent Council meeting while Committee Chairpersons were being selected, I opted to chair the Journals and Curriculum Committees. We have provision to co-opt more members. We plan to frame the new Terms of Reference for evaluation and recognition of journals bringing it at par with Higher Education Commission. We wish to improve their standards and look into the possibilities how to train the editors to improve the quality of manuscripts accepted for publication as well as improving the standard of the Journals.

The organizers also honoured three senior medical editors in recognition of their contribution to promote the discipline of medical journalism in Pakistan. Those who were selected for this honour included Dr.Fatema Jawad Chief Editor, Journal of Pakistan Medical Association, Maj.Gen. Muhammad Aslam Pro Vice Chancellor of National University of Medical Sciences (NUMS) and Mr.Shaukat Ali Jawaid Chief Editor Pakistan Journal of Medical Sciences who is also Secretary EMAME. Citations read on this occasion gave details of their contributions and accomplishments in his field over the years.

**Prof. Nasir Khokhar** outgoing President of PAME chaired the General Body Meeting. Dr. Irfan presented the secretary’s report while Mr.Shaukat Ali Jawaid presented the Treasurer’s report. All the office bearers were elected unanimously. Dr. Jamshed Akhtar took over as the new President while Dr. Irfan and Dr.Sina Aziz were elected as President-elect and General Secretary. Prof. Nazeer Khan was elected as Joint Secretary/Treasurer. Prof. Col.Alamgir Khan, Prof.Saira Afzal, Dr.Masood Jawaid and Prof. Ayub were elected as members of the Executive Council.

## Second Session

This session was moderated by Dr. Irfan. **Prof. M. Alamgir** Editor of Pakistan Armed Forces Medical Journal talked about peer review, its importance and challenges. Peer review, he pointed out, was the biggest hurdle in publication and at times a nightmare for the editors. PM&DC as well as HEC focus on peer review. He also talked about the trust of the readers, peer review benefits the Editor, authors, reviewers, science and society at large as it improves the quality of the manuscripts before they are published. He referred to the paucity of qualified reviewers and training of reviewers which are a time consuming process. Every journal must have its own Reviewers database and arrange for training of reviewers. Quality of the reviews was also important. He suggested that PAME must organize workshops for training of reviewers and training courses for Reviewers, Editors. He also talked about offering incentives to the reviewers, recognition of reviewers, deadlines for review, developing a system, reviewers hunting, and accountability of reviewers.

Research is an important weapon which should be used for benefit of human beings-Nasiruddin Azam Khan

**Prof. M. Ayub** Chief Editor Kashmir Journal of Medical Sciences highlighted the Peer Review implications in Online Journal System. Peer review, he said, was a critical review by an expert in that particular field. It helps improve quality and standard of articles before publication. New Open Journal System, he said, has the option of single, double blind and open peer review. The system sends automatic reminders after the due date but the problem is that some of our authors as well reviewers and some faculty members are not computer literate. He suggested promoting the culture internet use among the reviewers, training opportunities for reviewers, besides training of reviewers to use Open Journal System. He further suggested that some honorarium should be paid to the reviewers. HEC can help finding qualified reviewers. We all need to update our knowledge. Regulatory bodies like HEC and PM&DC should encourage the editors rather than creating problems for them. He also suggested that PAME can start its own accreditation system for the medical journals which should be based on transparent criteria.

**Dr. Shahbaz Ali Khan** discussed authorship issues. Since the number of authors has increased, now many journals, he stated, also require details of individual author’s contribution. Addition or deletion of authors is finally decided by the Editor.

**Prof. Khalid Mahmood** Editor of JPMI spoke about plagiarism and highlighted the importance of ethics in medical writing. He also referred to academic dishonesty, pressure to publish besides different types of plagiarism. Source of funding and conflict of interest must be disclosed by the authors, he added.

**Dr. Mian Saad Ahmad** gave an overview of indexation of Pakistani medical journals. Most of the journals being published from Pakistan, he said, were quarterly. Only three journals have got Impact Factor. Twenty eight journals have functional websites on the net, thirty nine journals have given their peer review policy, two journals practice open peer review system. Six journals have female Chief Editors, a total of 5,524 articles were published in all the journals during 2017 and almost 80% of the manuscripts published were original articles.

**Dr. Mohammad Irfan** in his brief presentation stated that model of peer review was a key element. He also talked about different peer review systems.

**Prof. Z. A. Bhutta** a noted Pediatrician and researcher in his presentation talked about Pakistan’s low contribution to the world medical literature. The only incentive for research in Pakistan, he stated, seems to be for promotion. We give too much importance to quantity rather than paying attention to quality of research. Every institutions is now publishing a journal but it is the culture of quality which matters. He also talked about the process of Evaluation and said that overseas while taking decisions for promotion and selection, the candidates are asked to submit their best five papers which are then evaluated. We must give importance to quality. Only a few scientists have made tremendous contribution to science which has pushed our numbers tremendously as most of the well cited Publications are from those three scientists. We need to change the system of evaluation of science and scientists and promote quality research culture. HEC, PM&DC, Pakistan Science Foundation should all give importance to quality. He also referred to the international criteria of Evaluation of scientists like h Index and i10Index etc. Prof. Bhutta suggested that the editors of medical journals must raise the bar of quality of articles being accepted for publication. Unfortunately, we have a false sense of achievements in Pakistan. Research takes time and teaching and training in research methodology should start at the undergraduate level. Supervisors, Heads of the Departments should not abuse their position. It is the institutions who should decide as to who will be the first author. Students and postgraduates need to be protected by the institutions, Prof. Bhutta remarked.

Pakistan needs a National Commission on Biomedical Sciences to identify types of research needed- Daud Khan

We need to change the system of evaluation of science and scientists and promote quality research culture- Z.A.Bhutta

## Afternoon Session

**Prof. Saeed Baig** was the first speaker in the afternoon session who discussed the pros and cons of medical writing. Authors, she stated, look for short cut and fast publication. They lack writing skills, have no proper training in medical writing. Authors also find browsing lethargic, they have no patience and have fear of writing. All this then leads to Gift authorship and ghost submissions to many journals. Institutions, she felt, should take the responsibility of providing the faculty members facilities and training opportunities.

**Prof. Nazeer Khan** Editor of JSMUJ talked about citation indexes. Junior and new researchers, he opined, will have low h Index. Self-citation also increases h Index, he added.

**Dr. Zafar Ali** discussed violation of publication ethics and mentioned about plagiarism, dual publication, and duplicate submissions. The solution lies in taking action against all those who indulge in scientific misconduct, he remarked.

**Figure:**
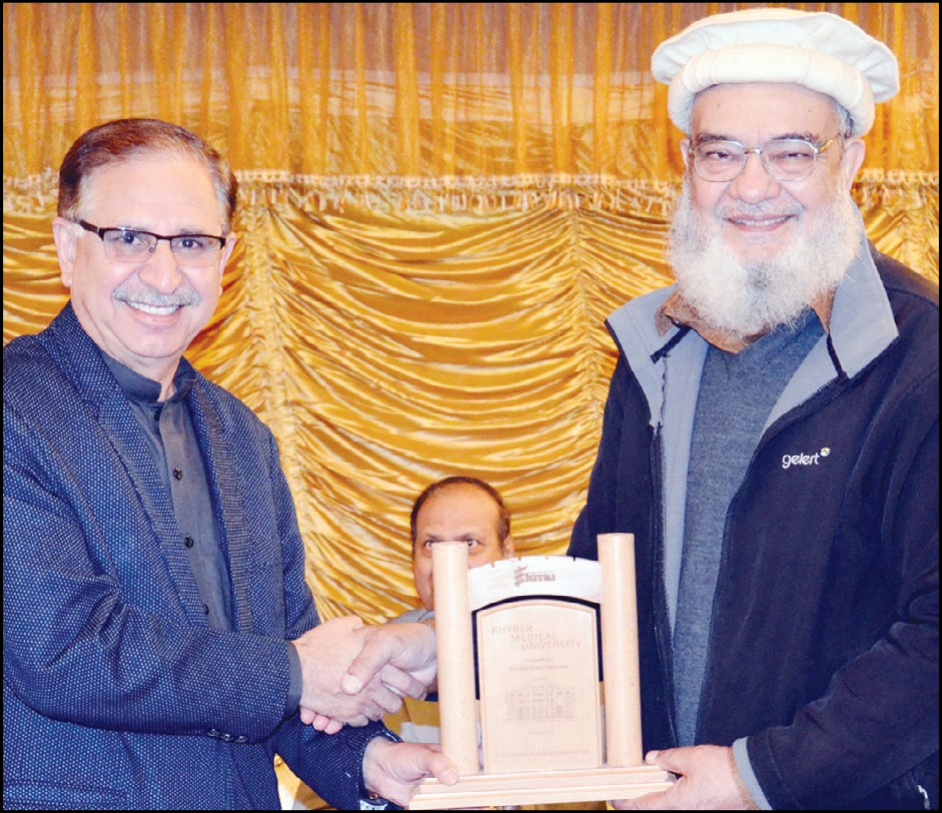
Khyber Medical University Journal celebrated its 10th year of successful publication during the Fourth National Conference of Pakistan Association of Medical Editors held at Peshawar recently. Picture sows Prof. Arshad Javed VC KMU presenting a Memento to Prof. Lutfullah Kakakhel former VC of KUST who was the chief guest at this occasion.

## Challenges for Pakistani Journals

**Prof.Saira Afzal** Editor of Annals of King Edward Medical University moderated this Open House session wherein different issues and challenges faced were discussed and possible solutions highlighted by the participants. She highlighted the importance of having proper SOPs and all review process should be documented. It was also stated that medical institutions need to have a statistician on their staff, challenges faced with Open Journal System and other manuscript management systems were also discussed. Other issues which came under discussion included citation analysis, types of articles, role of HEC and PM&DC. It was agreed that qualitative research should be preferred. **Dr. Usman Mehboob** a medical educationist remarked that research should be fit for the purpose of specific study. Design of the study also matters. Our researchers do not cover social issues, he remarked.

## Decade of successful publication of KMUJ

Ten Years of successful publication of Khyber Medical University Journal was a part of the Fourth National Conference of Pakistan Association of Medical Editors hosted by the Khyber Medical University from March 3-4^th^2018. **Prof. Lutfullah Kakakhel** former Vice Chancellor of Kohat University of Medical Sciences was the chief guest on this occasion. In his speech he said that we need to introduce modern technology in medical journals to keep pace with the developments taking place at a fast pace. All the journals, he further stated, should go on Open Journal System or other similar programmes which offer numerous advantages and accelerates the whole process of Publications.

Prof. Lutfullah Kakakhel who is an expert in Information Technology, it may be mentioned here had helped start using OJS for their journal from the very first day. He also conducted an orientation course for the editors of medical and science journals at Higher Education Commission many years ago to acquaint them with the OJS. He also showed a small gadget which he said has large data base including thousands of articles, over four hundred books and numerous other features which are useful for the researchers.

PAME can start its own accreditation system for the medical journals which should be based on transparent criteria- Prof. Ayub

**Prof. Akhtar Sherin** Chief Editor of Khyber Medical University Journal speaking at the occasion said that they started this journal in 2008 as KUST Medical Journal and later it was renamed as Khyber Medical University Journal. Prof. Lutfullah Kakakhel was the moving spirit behind this venture and we started using OJS from the very first day. Prof.Tariq Mufti had joined us as Principal of the Medical College and we also benefitted from his experience as he had been working as Chief Editor of Journal of Ayub Medical College, Abbottabad. Terms of Reference for running the journal were approved by the Board of Studies and maintaining quality was our aim. Unfortunately soon after, Prof. Lutfullah Kakakhel was kidnapped by the Taliban and we ran into trouble. When the journal was published it was also the last day of Prof.Tariq Mufti and we hosted a farewell lunch for him. He also highlighted the problems they had to face. Prof. Hafeezullah and Prof. Saleem Khattak helped us a lot. Prof.Arshad Javed the present Vice Chancellor of KMU, he further stated, has now given complete freedom and autonomy to the Chief Editors to run and manage their respective journals and there is no interference by the administration at all.

KMU VC has given complete independence to Chief Editors to run and manage their respective journals-Akhtar Sherin

**Prof.Tariq Mufti** in his speech on the occasion recalled his efforts for Journal of Ayub Medical College. He also paid tributes to late Dr. Muhammad Ilyas who was a qualified, competent cardiologist but he was neglected by his colleagues. He was keen researcher and he encouraged me to write. He also commended the encouragement and support he had from Prof.A.J.Khan the founder Principal of Ayub Medical College, Abbottabad.

**Mr.Shaukat Ali Jawaid** Chief Editor Pakistan Journal of Medical Sciences highlighted the contributions made by late **Prof.Tahir Saeed Haroon** to promote the discipline of dermatology and earn respect for dermatologists in the society and recognition for this specialty. The occasion was used to offer Fateha for the departed soul who also served as President of Pakistan Medical Journalists Association which was later renamed as PAME. He had contributed a lot to promote medical journalism in the country as well.

**Figure:**
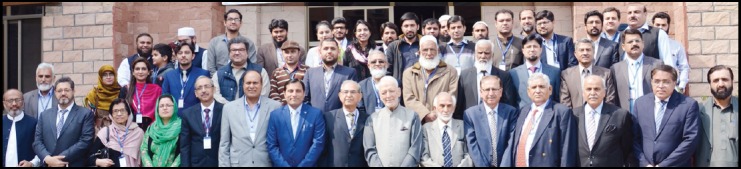
Participants to the PAME's Fourth National Conference held at Khyber Medical University Peshawar on March 3, 2018 photographed alongwith Prof. Nasiruddin Azam Khan (Chief Guest) Prof. Daud Khan (Guest of Honour), Prof. Arshad Javed VC KMU and executive of PAME.

**Dr. Jamshed Akhtar** who took over as the new President of PAME in his speech highlighted the importance of maintaining standard and quality of manuscripts accepted for publication. Recently HEC, he said, has derecognized twenty journals. We need to move forward, give due emphasis to logic and philosophy. Efforts should be made that all our medical journals move to W category of the HEC recognized medical journals, he remarked.

**Prof.Arshad Javed** Vice Chancellor of KMU said that in the new set up in PM&DC he has been given the responsibilities to chair the Curriculum and Journals Committee. We need to change the rules of PM&DC, revise the evaluation criteria for medical journals and this is a rare opportunity to make some important changes and bring the PM&DC Journals Evaluation criteria at par with that of HEC. We have provision and Vice Chancellors of various universities have nominated three editors to this committee, hence with their input, he was hopeful of making some positive change, he added.

PM&DC is planning to frame new Terms of Reference for Evaluation and Recognition Of biomedical journals-Prof. Arshad Javed

## Post Conference Workshops

On Day–2 of the conference four workshops were organized on Epidemiology Study Designs and Basic Biostatistics, Essential of Scientific Manuscript Submission to Medical Journals, Online Journal Management System, and Peer Review: Theory and Application.

